# Validation of the Persian Version of the Langer Mindfulness Scale

**DOI:** 10.3389/fpsyg.2017.00468

**Published:** 2017-03-30

**Authors:** Fatemeh Moafian, Francesco Pagnini, Hooshang Khoshsima

**Affiliations:** ^1^Department of English, Chabahar Maritime UniversityChabahar, Iran; ^2^Department of Psychology, Harvard UniversityCambridge, MA, USA; ^3^Department of Psychology, Catholic University of MilanMilan, Italy

**Keywords:** ellen langer, langerian mindfulness, langer mindfulness scale, confirmatory factor analysis, scale validation, cross-cultural psychology

## Abstract

This study validates the Persian version of the Langer Mindfulness Scale (LMS). The original scale consists of 21 items and 4 subscales; namely, novelty producing, novelty seeking, engagement, and flexibility. In this study, four samples including 2271 individuals in total participated. Confirmatory factor analysis was employed to test the factorial structure of the Persian version. The results verified a two-factor structure including novelty producing and novelty seeking for the scale and the two subscales of engagement and flexibility were omitted due to marginal fit. The questionnaire showed satisfying psychometric properties in terms of reliability. Furthermore, convergent and discriminant validity of the instrument was examined via investigating the relationship between the Persian LMS with the WHOQOL instrument and negative and positive affect scales. The findings revealed a significant positive relationship between the Persian LMS and positive affect, physical health, psychological health and environmental health. No significant correlations were found between the LMS, social relationships and negative affect.

## Introduction

Mindfulness is a multifaceted and complex concept that has been investigated by scientists for the last 40 years (Pagnini and Phillips, 2015). There are two main approaches to mindfulness, namely Eastern and Western. The Eastern approach is rooted in Theravada Buddhism and it was westernized by Kabat-Zinn (1990). It focuses on present-based attention and a non-evaluative perspective, and it is closely related to the practice of meditation. While in harmony with fundamental tenets of Buddhist-based mindfulness, the Western approach provides a different framework for understanding and achieving mindfulness. It has been developed by Langer (1989); Langer et al. (1978), and it considers mindfulness as the process of drawing novel distinction. In the current paper, we will refer to this latter definition of mindfulness.

As reality is always in constant change, being in the present moment focuses on the act of noticing big and subtle changes (Langer and Moldoveanu, 2000). When mindful, people are sensitive to the context and the environment, they welcome novelties, they create new categories for structuring perception, and they present multiple perspectives in problem solving (Langer and Moldoveanu, 2000). The awareness of multiple perspectives helps to reduce the need for previously established categories, in effect promoting mind-openness (Langer, 1992). Some of those categories deal with judgment, for example “bad” and “good.” A mindful perspective facilitates the realization that an event is not “good” or “bad” in itself. It always depends on the individual's point of view (Langer, 1989). *Mindlessness* is the reverse of mindfulness, consisting of relying upon previously established categories. When one is mindless, (s)he transforms into a pre-programmed machine that behaves according to past memories. In this case the person is entangled in a single, inflexible perspective, unaware of other possible ways of knowing. Being mindless leads to rule-governed and law abiding behavior while mindfulness leads us toward a behavior guided by rules and laws, but not predetermined by them (Langer, 2000).

Principal components of mindfulness include novelty seeking, novelty producing, flexibility and engagement (Pirson et al., 2012). Novelty seeking is the proneness to be curious and open toward the environment and oneself. Novelty producing refers to the ability and the tendency of a person to create new categories, with innovation and creativity, rather than relying on previous categorizations. Flexibility is the ability of considering experiences from multiple perspectives, resulting in a better adaptation to the environment. Engagement is the attitude toward an active interaction with the environment, when the person is likely to notice subtler details and changes in social/environmental context.

There are several benefits related to mindfulness, spanning from health and well-being to business and artistic endeavors (Phillips and Pagnini, 2014). People who report higher mindfulness tend to have higher quality of life and psychological well-being (Langer, 1989; Pagnini and Langer, 2015) even in the case of severe health conditions (Pagnini et al., 2014, 2015, 2016). Mindfulness also appears to be connected to longevity (Alexander et al., 1989). In general, mindfulness is related to better performances in many fields, including education (Langer, 2000), entrepreneurship (Rerup, 2005) and leadership (Sauer and Kohls, 2011), music performance (Langer et al., 2009) and sports activity (Kee and Wang, 2008).

Several questionnaires have been devised to measure mindfulness, but they generally refer to a different concept, the Eastern mindfulness concept (Kabat-Zinn, 1990). Examples of these questionnaires include the Freiburg Mindfulness Inventory (FMI; Walach et al., 2006), the Mindfulness Attention Awareness Scale (MAAS; Brown and Ryan, 2003) and the Five Facet Mindfulness Questionnaire (FFMQ; Baer et al., 2006).

There is currently only one tool that assesses the construct of mindfulness, as originally proposed by Langer (Haigh et al., 2011), the Langer Mindfulness Scale (LMS; Pirson et al., 2012). The LMS is self-report questionnaire written in English, consisting of 21 items that cover all the four main elements of mindfulness: novelty producing, novelty seeking, engagement, and flexibility (Langer, 2004). Each item is scored on a 7-point Likert scale, from 1 (strongly disagree) to 7 (strongly agree), with some reverse coded items. Overall scores range from 21 to 147, with higher scores reflecting higher mindfulness. The original version of the scale proved reliable psychometric validity (Pirson et al., 2012). The scale is widely used to assess mindfulness and has been translated and validated into Malaysian (Leong and Rasli, 2013), German (Haller, 2015), and Italian (Pagnini et al., in preparation), with other validations pending, including Indian, Sweden, Chinese, and Greek versions. When translated and validated, the LMS was modified to fit the collected data and to adapt to local cultures and customs. For example, the Malaysian version includes only two factors and the German one contains merely one factor.

Due to the specific characteristics and the relevance of Langer's approach to mindfulness, it seems relevant that the LMS could be translated and validated cross-linguistically and cross-culturally so that other communities can take advantage of it. Accordingly, this current study attempts to analyze the validation of the LMS in Persian language. In other words, the goal of the research is to investigate the psychometric properties of the Persian LMS including content and construct validity as well as reliability analysis. Moreover, discriminant and convergent validity of the scale are investigated. It is worth noting that to check the discriminant and convergent validity of the scale, the associations between the LMS and discriminant (negative affect) and convergent constructs (positive affect, the quality of life) are investigated. Owing to the fact that the LMS is not comparable with the other mindfulness scales as it is based on information processing and creativity theory (Haigh et al., 2011) and that there is a notable distance between Western and Eastern mindfulness (Sauer et al., 2013), other constructs that had correlations with the original LMS and its other translations were chosen to test discriminate/convergent validity. To this aim, on the basis of the previous studies (e.g., Haigh et al., 2011; Pirson et al., 2012) on different versions of the LMS, Positive Affect Scale, World Health Organization Quality of Life Instrument and Negative Affect Scale were selected.

In the end, it is worthy of note that there are several cultural differences between Iran and the U.S., which may influence the process of mindfulness assessment. Specifically, according to Hofstede's cultural dimensions (Hofstede et al., 1991; Javidan and Carl, 2004), Iran is considered a collectivistic society while U.S. is an individualistic one. Moreover, there are some differences between Iran and U.S. regarding uncertainty avoidance, long term orientation and the level of indulgence. These may lead to different codes of beliefs and behaviors in the two countries. These elements draw the cultural contexts in the two countries in which the expression of mindfulness may be different. Accordingly, it is hypothesized that the factor structure of the LMS would not be replicated in the Persian version of the scale.

## Method

### Participants

Four samples including 2271 participants in total took part in the current research. The first (*N* = 1200), second (*N* = 91) and third (*N* = 830) samples were recruited in the content and construct validity part and the fourth sample (*N* = 150) was recruited in the discriminant and convergent validity part. The participants included both male and female as well as both married and single individuals. Their age varied from 14 to 75 and they had different levels of education, from secondary school to Ph.D. The precise demographic information of the samples is depicted in Table 1.

**Table 1:** **The demographic profiles of the samples**.

		**Sample 1**		**Sample 2**		**Sample 3**		**Sample 4**	
		***N***	**%**	***N***	**%**	***N***	**%**	***N***	**%**
Gender	Female	652	54.33	43	47.25	415	50	83	55.33
	Male	539	44.91	48	52.74	408	49.15	64	42.6
	Unknown	9	75	0	0	7	84	3	2
	Total	1200	100	91	100	830	100	150	100
Level of education	Under diploma	29	2.41	0	0	86	10.36	2	1.33
	Diploma	110	9.16	0	0	180	21.68	15	10
	A.A. holders or students	59	4.91	2	2.19	90	10.84	13	8.66
	B.A/B.S. holders or students	623	51.91	19	20.87	342	41.2	61	40.66
	M.A./M.S. holders or students	131	10.91	54	59.34	86	10.36	37	24.66
	Ph.D. holders or students	54	4.5	14	15.38	18	2.16	14	9.33
	Holders or students of other university degrees	141	11.75	1	1.09	5	0.6	0	0
	Unknown	53	4.41	1	1.09	23	2.77	8	5.33
	Total	1200	100	91	100	830	100	150	100
Marital status	Single	861	71.75	52	57.145	519	62.53	116	77.33
	Married	315	26.25	35	38.46	275	33.13	26	17.33
	Unknown	24	2	4	4.39	36	4.33	8	5.33
	Total	1200	100	91	100	830	100	150	100
Age	Range	16–75		19–50		14–64		18–56	
	Mean	24.57		29.04		25.48		26.72	
	SD	7.53		6.41		7.63		5.59	

### Instruments

#### LMS

Langer Mindfulness Scale (LMS) consists of 21 items and four subscales of novelty producing, novelty seeking, engagement, and flexibility (Langer, 2004). Six items in the instrument are associated with novelty producing, six with novelty seeking, five with engagement, and four with flexibility. The respondents answer the items on a seven-point Likert scale ranging from strongly disagree (1) to strongly agree (7). The total reliability of the instrument, calculated via Cronbach's alpha, was found to be 0.83 and the reliability of the subscales were 0.83,0.74,0.63, and 0.54 for novelty producing, novelty seeking, engagement and flexibility, respectively (Bodner and Langer, 2001). Psychometric properties of the original LMS included CFI fit indices ranging from 0.92 to 0.95 and an RMSEA of 0.052 to 0.063 (Pirson et al., 2012). The scale has been translated and validated into different languages including Malaysian (Leong and Rasli, 2013), German (Haller, 2015), and Italian (Pagnini et al., in preparation) with satisfying psychometric properties. For example, all three versions showed high internal consistency (Cronbach's alpha of 0.82 for the German version, 0.78 for the Malaysian one, 0.83 for the Italian LMS).

#### Positive affect scale

The Positive Affect Scale, designed by Watson et al. (1988), includes 10 items evaluating respondent positive affect. The participants demonstrate their different feelings during the past few weeks via responding to questions posed on a five-point Likert scale ranging from never (1) to always (5). The reliability of the positive affect measure was found to be 0.88 for the English version (Watson et al., 1988). In the current study, the Persian version of the questionnaire was applied. According to Bakhshipour and Dozhkam (2006), the scale enjoys acceptable reliability and validity in Persian language. In this study, the reliability of the questionnaire, calculated via Cronbach's alpha, was found to be 0.83.

#### World health organization quality of life instrument (WHOQOL)-BREF

The WHOQOL-BREF consists of 26 items including four domains of physical health, psychological health, social relationships, and environmental health. The total number of the items for the four domains is 24; seven items are associated with physical health, six with psychological health, three with social relationship and eight with environmental health. There are also two items concerning overall quality of life and general health. The respondents are required to answer the items on a five-point Likert scale about the quality of their life in the last 4 weeks. The total reliability of the instrument and the reliability of the four subscales were reported to be between 0.73 and 0.89 for the version written in English (WHOQOL Group, 1998). In the present study, the Persian version of the questionnaire was employed. According to Nejat et al. (2006), the Iranian-translated instrument has good psychometric properties. The total reliability of the instrument in the present study, calculated via Cronbach's alpha, was 0.87. The reliability of the subscales were 0.69, 0.74, 0.72, and 0.27 for physical health, psychological health, environmental health and social relationships, respectively. The low reliability of the latter social relationships subscale maybe due to the fact that the researchers had to omit one of the three subscale questions, an inquiry into sexual satisfaction, since the participants were both single and married. This reduction in subscale questions may plausibly justify the low reliability value.

#### Negative affect scale

The Negative Affect Scale, designed by Watson et al. (1988), is composed of 10 items examining the negative affect of the participants. The participants show their different feelings during the past few weeks via responding on a five-point Likert scale ranging from never (1) to always (5). The reliability of the negative affect was found to be 0.87 for the English version (Watson et al., 1988). In the present research, the Persian version of the questionnaire was employed. According to Bakhshipour and Dozhkam (2006), the scale enjoys acceptable reliability and validity in Persian language. In this study, the reliability of the questionnaire was 0.85, calculated via Cronbach's alpha.

### Procedure

Regarding the first sample of the study, the data was collected from the participants in two cities (i.e., Mashhad and Chabahar) in Iran. Concerning the second, third and fourth samples, the data was collected only in Mashhad. The participants were asked to take the questionnaires, fill them out and immediately returned them to the researchers. Concerning ethics approval, the current study is exempt from this requirement since in the context where the study was carried out when data collection process does not harm participants neither physically nor mentally considering only passive consent—including “not opting out or not objecting to the study” (Dornyei, 2007, p. 70)—is enough. To obtain reliable data, the participants were assured that the anonymity considerations would be observed; that is, the identity of the participants would be kept from everyone, including the research via coding the questionnaire numerically.

To assess the content validity of this instrument, the forward and back translation process was employed. In doing so, three experts in the fields of psychology, education and applied linguistics who knew both English and Persian were asked to translate the English version of the scale into Persian. The received translations were compared with each other as well as with the original version. Out of the three translations, one was finalized. The finalized version was retranslated into English by another three experts majoring in the field of applied linguistics. These experts had not seen the English version of the scale. The comparison between the back translations and the English revealed that the back translated versions agreed with the English one. Then, the Persian scale was shown to a judge who was a psychologist, an expert in mindfulness, and knew both English and Persian well. According to his comments, some modifications were made on the Persian version.

The next step was the recruitment of the participants. Prior to recruiting, the questionnaire was provided to several people to read. These individuals were asked to specify if they encountered any comprehension problems of individual questionnaire items. No issues were indicated; consequently, the recruitment of the participants commenced. 1,200 persons were asked to fill out the questionnaire. Before conducting any analysis, the reliability of the data was checked using Cronbach's alpha. The total reliability of the instrument was acceptable (*r* = 0.71). However, the reliability of the subscales was not satisfactory especially for the two subscales of Engagement and Flexibility (r_NP_ = 0.71; r_NS_ = 0.50; r_E_ = 0.31; r_F_ = 0.29). The possible reasons were scrutinized. According to the participants' feedback, data scrutinization, as well as through consultation with a psychometrician, it was concluded that there were still some problems in the translation of the questionnaire. Accordingly, several modifications were done on the Persian tool. The researchers altered the questions by replacing ambiguous words with ordinary words which were more familiar for the participants from different levels of society, and were closer in equivalency to the words utilized in English version. Simpler word arrangements were also used and the complex ones were broken down into unambiguous syntaxes. Via this process the translation improved considerably. Once again, three judges (two psychometricians and one cognitive psychologist) were asked to evaluate the content and the translation of the instrument in terms of relevance, clarity, simplicity, ambiguity and culture fit (Yaghmale, 2003). The three judges knew both English and Persian languages as well as having a great deal of experience in validating instruments from other languages into Persian. They corroborated the quality of the translation. To ensure the preservation of the content of the original scale in the translated one following several modifications, the authors asked a psychologist who knew both Persian and English well to back-translate the last Persian version into English. The results of back-translation were similar to the English version.

The obtained Persian scale was piloted on the second sample with 91 subjects. The reliability analysis was carried out on the data using Cronbach's alpha. The total reliability of the questionnaire was 0.77 which showed an improvement over the previous one. Cronbach's alpha if item deleted option was also run to find the problematic items. The findings revealed that items 7, 8, and 12 were problematic and their omission would lead to increased reliability (with the deletion of 7, *r* = 0.79; with the deletion of 8, *r* = 0.78; with the deletion of 12, *r* = 0.80). The reliability of the subscales was also computed. The reliability of the subscales including novelty producing, novelty seeking, engagement and flexibility were 0.89, 0.62, −0.04, and 0.20, respectively. Compared with the reliability measures in sample 1, the reliability of the first two subscales had increased; however, the reliability of the last two factors had decreased. It's worth mentioning that the problematic items mentioned above (items 7, 8 and 12) were located on the last two subscales possessing low reliability. Items 7, 8, and 12 were examined and once again some modifications were done on their translation to determine whether the reliability of the instrument would increase without their omission. In this way, the instrument was prepared to be administered on the main sample.

The third sample was the major of the study. Eight hundred and thirty participants completed the questionnaire. Via Cronbach's alpha, the total reliability of the questionnaire and the reliability of subscales were checked. The total reliability of the questionnaire was 0.73. The results of Cronbach's alpha from “scale if item deleted” option revealed that the problematic items were 7, 8, 12 (with the deletion of 8, *r* = 0.755; with the deletion of 12, *r* = 0.747; with the deletion of 7, *r* = 0.742). They were the same problematic items indicated during the previous sample. It seemed that modifications on translation could not solve the problems of these items and something else appeared to be wrong with them. The problem might have been within the content of these items and it appeared they were not clear and specific enough for the participants to decide upon the correct answer. Accordingly, the items were deleted and the total reliability, calculated via Cronbach's alpha, increased to 0.78. The reliability of the subscales was also assessed. The reliability of the novelty producing measure was 0.77. The problematic item in this subscale was 14. A reliability value of 0.78. was achieved through its deletion. The reliability of novelty seeking was 0.55 and there was no problematic item in this subscale. The reliability of engagement with the three remaining items (items 4, 15, 19) was 0.31; the problematic item in this subscale was 4 which its deletion led to the increase of reliability to 0.39. However, since the value of Cronbach's alpha was under the acceptable range >0.40 (Simon, 2005; cited in Ostovar, 2012), the subscale was deleted. The reliability of flexibility with the three remaining items (items 3, 11, 16) was found to be 0.32 and there were no problematic items in this subscale. Due to the same issue with the engagement subscale, the flexibility subscale was also deleted.

The finalized Persian version of Langer Mindfulness Scale with two remaining subscales and 11 items was prepared for confirmatory factor analysis (CFA).

### Data analysis

Confirmatory factor analysis (CFA) and reliability analyses were conducted to investigate the construct validity and reliability of the instrument. To this aim, Amos 19 and SPSS v 22 were employed. The level of significance was set at 0.05.

CFA was conducted to verify the factorial structure of the translated version of the questionnaire. To compute the fit indices including the ratio of chi-square to degrees of freedom (χ^2^/df), Tucker-Lewis Index (TLI), Comparative Fit Index (CFI), Normed Fit Index (NFI), Incremental Fit Index (IFI), Goodness-of-Fit Index (GFI), Adjusted Goodness of Fit Index (AGFI), the Root Mean Square Error of Approximation (RMSEA), and Standardized Root Mean Square Residual (SRMR), structural equation modeling with ML estimation was applied.

The internal consistency of the questionnaire along with the internal consistency of the subscales was estimated via Cronbach's alpha coefficients. Additionally, the inter-correlations among the subscales were assessed via the Pearson correlation coefficients.

Pearson product moment correlation was employed to examine discriminant and convergent validity of the scale.

## Results

### Construct validity

**Figure 1:**
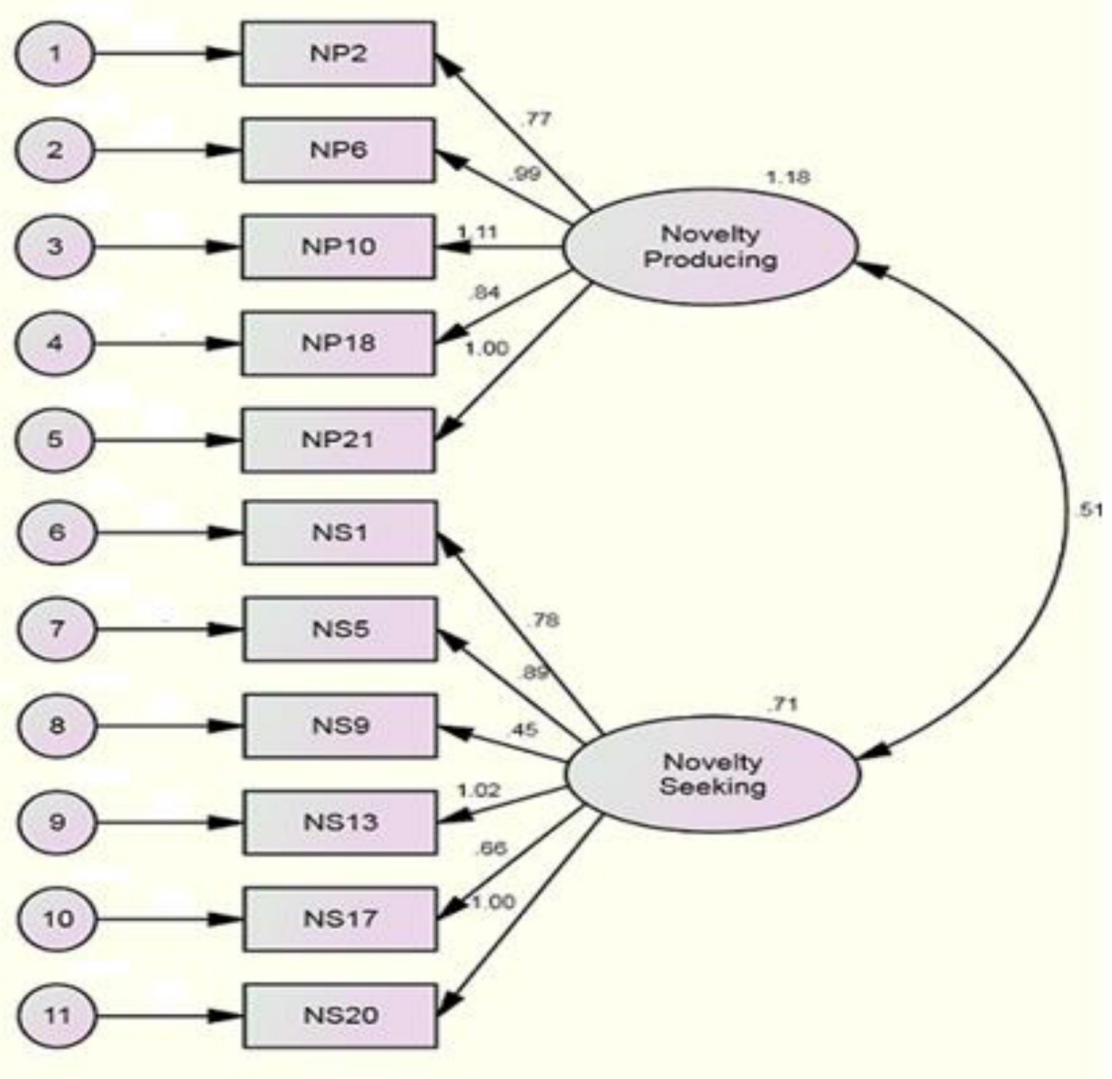
**The Two factor path diagram for Model 1**.

To verify if the obtained factorial structure suggested a good fit to the data, CFA was conducted. In doing so, Amos 19 software was employed, using structural equation modeling with ML estimation. The fit indices including χ^2^/df, TLI, CFI, NFI, IFI, GFI, AGFI, RMSEA, and SRMR were considered to check the model. The obtained values for the intended indices for Model 1 were as follows: χ^2^ = 311.08 (df 43), TLI = 0.81, CFI = 0.85, NFI = 0.83, IFI = 0.85, GFI = 0.93, AGFI = 0.89, and RMSEA = 0.08. The results of CFA are depicted in Table 2 and Figure 1.

**Table 2:** **Confirmatory factor analysis for Models 1 and 2**.

**Fit indices 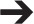 **	**χ^2^/df**	**TLI**	**CFI**	**NFI**	**IFI**	**GFI**	**AGFI**	**RMSEA**	***p***
Model 1	311.08	0.81	0.85	0.83	0.85	0.93	0.89	0.08	<0.01
Model 2	268.10	0.82	0.87	0.85	0.87	0.94	0.90	0.09	<0.01

Considering the values of fit indices in Table 2 for Model 1 and the magnitudes of the relationship of the items with their related subscales, it was decieded to omit item 9 to check the changes in the values of fit indices and to see whether a better model fit would be obtained. The reason why item 9 was deleted was the fact that the amount of its relationship with its subscales was low in comparison with those of other items. Consequently, item 9 was omitted and Model 2 was ready for CFA.

**Figure 2:**
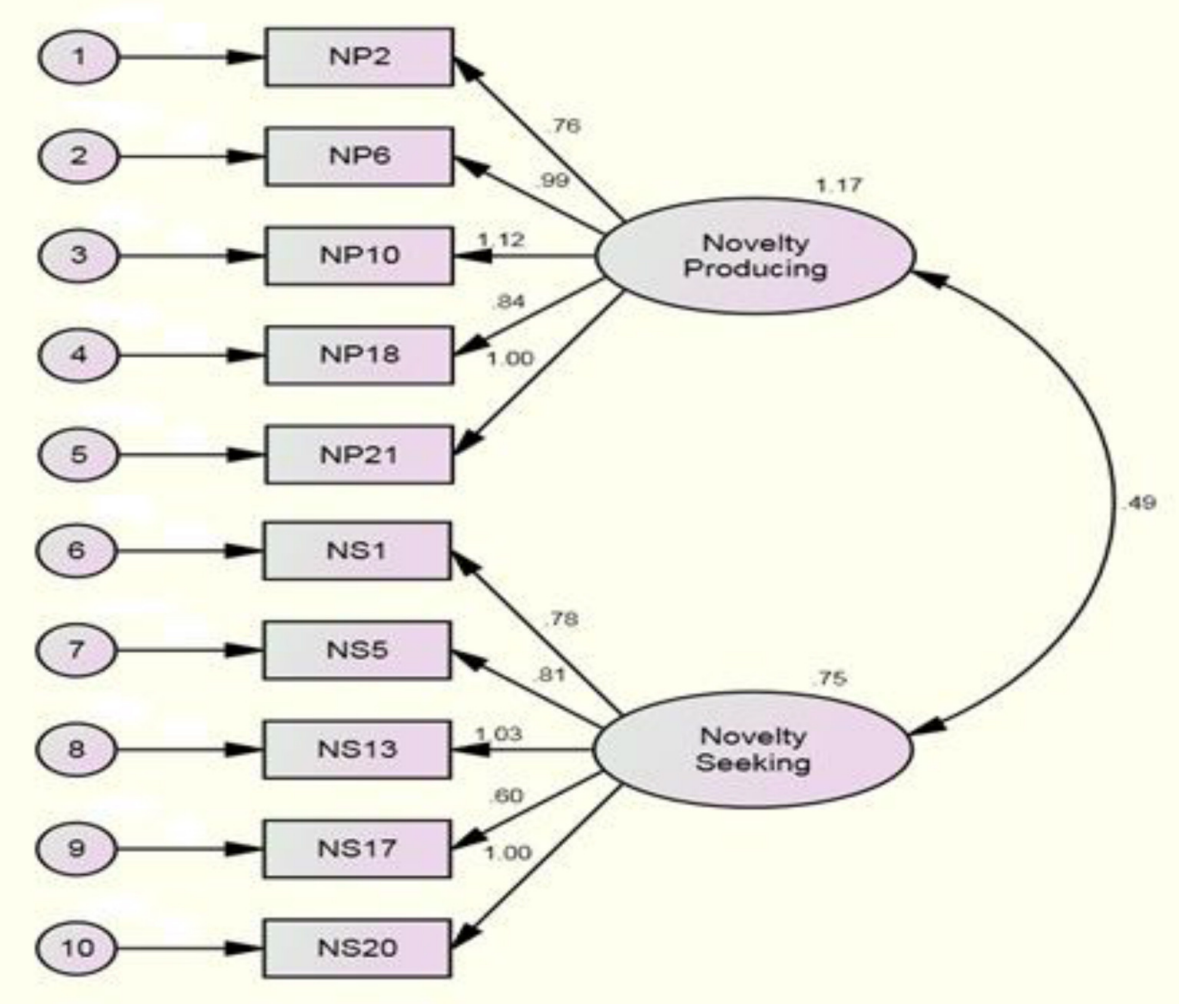
**The Two factor path diagram for Model 2**.

The results of CFA for Model 2 are illustrated in Table 2 and Figure 2. As the measures of fit indices for Model 2 revealed, all fit indices had increased except for RMSEA (χ^2^ = 268.10 (df 34), TLI = 0.82, CFI = 0.87, NFI = 0.85, IFI = 0.87, GFI = 0.94, AGFI = 0.90, and RMSEA = 0.09). Therefore, it was concluded that Model 2 could support the construct validity of the questionnaire more appropriately. That is, the construct validity of the instrument with two subscale and 10 items was supported.

### Reliability analysis

To assess the reliability of the questionnaire as well as the inter-correlations between the two subscales, SPSS v 22 was employed. The total reliability of the scale, estimated via Cronbach's alpha, was found to be 0.76. The internal consistency of the two subscales, computed via Cronbach's alpha, were 0.78 and 0.55 for novelty producing and novelty seeking, respectively (see Table 3); the achieved magnitudes were within the acceptable range (Gardner and Gardner, 2012).

**Table 3:** **The factor loading and reliability of the persian LMS**.

**Construct**	**Number of Items**	**Items**	**Loadings**	**Alpha**
Novelty Producing	5	NP 21	0.637	0.78
		NP 18	0.602	
		NP 10	0.819	
		NP 6	0.725	
		NP 2	0.481	
Novelty Seeking	5	NS 20	0.609	0.55
		NS 17	0.289	
		NS 13	0.578	
		NS 5	0.405	
		NS 1	0.470	
The Persian LMS	10			0.76

To determine the inter-correlations between the two subscales, Pearson correlation coefficient was calculated. The results revealed that there was a positive significant relationship between the two factors and the amount of the relationship was 0.44.

### Correlations

To check the discriminant and convergent validity of the scale, the associations between the LMS and discriminant (negative affect) and convergent constructs (positive affect, the quality of life) were investigated. As it was stated earlier, owing to the fact that the underlying theoretical framework of the LMS is different from that of the other mindfulness scales which are based on the Eastern mindfulness (Sauer et al., 2013), other correlated constructs with the original LMS and its other translations were selected to assess discriminate/convergent validity. To this end, the fourth sample (*N* = 150) was recruited and the participants were required to fill out the obtained Persian LMS and the Persian versions of the WHOQOL instrument and negative and positive affect scales.

The results revealed that there were statistically significant positive associations between the scores in the LMS and those in the positive affect (*r* = 0.49, *p* < 0.05), physical health (*r* = 0.29, *p* < 0.05), psychological health (*r* = 0.34, *p* < 0.05) and environmental health (*r* = 0.21, *p* < 0.05). However, no statistically significant correlations were found between mindfulness and social relationships as well as negative affect. That is, there was a weak positive relationship between mindfulness and social relationships (*r* = 0.14, *p* > 0.05) and a weak negative relationship between mindfulness and negative affect (*r* = −0.16, *p* > 0.05) (see Table 4).

**Table 4:** **The inter-correlations of the scales**.

	**1**	**2**	**3**	**4**	**5**	**6**	**7**
1. Mindfulness	–						
2. Positive Affect	0.49^**^	–					
3. Physical Health	0.29^**^	0.50^**^	–				
4. Psychological Health	0.34^**^	0.58^**^	0.71^**^	–			
5. Social Relationships	0.14	0.27^**^	0.40^**^	0.40^**^	–		
6. Environmental Health	0.21^**^	0.32^**^	0.62^**^	0.53^**^	0.47^**^	–	
7. Negative Affect	−0.16	−0.15	−0.32^**^	−0.43^**^	−0.22^**^	−0.33^**^	–

** *Correlation is significant at the 0.01 level*.

## Discussion

The aim of the current study was to examine the validation of the Persian version of the LMS. The original scale was written in English. Furthermore, it was initially designed and validated by Bodner and Langer (2001). The scale measured mindfulness through 21 items and four factors including novelty producing, novelty seeking, engagement and flexibilty.

In this research, the content, construct, convergent and discriminant validity and the reliability of the Persian version of the instrument were investigated. The content validity was assessed through experts' judgements in the field. The construct validity of the questionnaire was examined through CFA. The convergent and discrimenant validities were studied via the correlation of the scale with convergent (positive affect, the quality of life) and discriminant (negative affect) concepts; and reliability analysis was evaluated by way of Cronbach's alpha.

The results of CFA revealed that a 10-item scale with two factors (novelty producing and novelty seeking) could support the construct validity of the Persian version more appropriately than the 21-item instrument with four subscales (novelty producing, novelty seeking, engagement and flexibility). Accordingly, our hypothesis regarding the fact that the factor structure of the LMS would not be replicated in the Persian version of the scale was supported. The obtained results are comparable with the validation of the questionnaire by Haigh et al. (2011), the validation of the German version of the instrument by Haller (2015) and the validation of the Malaysian version of the scale by Leong and Rasli (2013) in that they didn't verify the original factorial structure of the scale suggested by Bodner and Langer (2001). The factorial structure proposed by Haigh et al. was a mono-dimensional model with 9 items and the one offered by Hamer was a single-factor structure with 6 items. The Malaysian version also supported a two-factor model. The acquired results (the omission of engagement and flexibility factors) in the current study might be plausibly justified by taking the following points into consideration: the first point is the fact that the content of some items were too broad or were arranged in a way that different interpretations could be extracted from them. For instance, item 15 is “I am rarely aware of changes.” The scope of the item was too broad for the participants to decide about. They could not determine changes to which aspects of their lives. Another example is item 8, I seldom notice what other people are up to, some of the participants interpreted it as being inquisitive and some understood it as meaning curiosity, which is an important feature for a mindful individual. It seems that some items on the original scale may need modifications. The other point worthy of consideration is the reality that although engagement and flexibility are important necessities of Langerian mindfulness, they may need to be operationalized with some other items, except the ones proposed by Langer in the LMS since as Sauer et al. (2013) declared, “mindfulness is likely to be a universal phenomenon that is dependent on culture and context” (p. 5).

The findings of convergent and discriminant validity indicated that there were statistically significant positive correlations between mindfulness and positive affect, physical health, psychological health and environmental health. However, no statistically significant correlations were found between mindfulness and social relationships or negative affect. The acquired findings confirm the results attained by Haigh et al. (2011) and Pirson et al. (2012) in that they found significant correlations between the LMS and positive affect as well as psychological and physical health. In regards to negative affect, inconsistent results were detected. Haigh and colleagues found mixed results, with inconsistent correlations across their samples; Pirson and colleagues also found significant negative associations between the two variables in two samples. The results of the current study for negative affect are similar to those found in the samples of the previous studies.

As it was stated earlier, mindfulness is the act of extracting new subtleties and differences that brings about higher sensitivity and awareness to situation and perspective, and finally superior control over life (Langer, 2000). It appears that a mindful person, by searching for new distinctions, could make his moment- to- moment experience as something new, preventing repetitive and dull experiences. The consequence of such a life-outlook is feeling happy and energized. Moreover, when people are open to new and various perspectives, they will have more options and alternatives for dealing with daily problems; this in turn may lead to more successes in life and less psychological pressures. Additionally, a mindful individual has better enhanced control over his or her life. This person welcomes and pioneers novel occurrences, and is no longer guided by past life experiences or association (Langer, 2000). There is little surprise that the outcome of such a life is possessing a high level of psychological health. Additionally, mind and body are interacting with each other and influencing one another's reactions; they are not distinct entities (Langer, 2009). Accordingly, higher levels of psychological heath might lead to superior physical health. The reason why no significant relationship was found between social relationships and the LMS may be due to the low reliability of this subscale as discussed in the instruments section, which in turn might influence the correlation between social relationship and the LMS. Otherwise, it is expected that a positive association would be found between the two variables due to the fact that social actions are mindfully reevaluated and interpreted by mindful people and they consider alternative explanations for what they encounter in their social interactions (Pirson et al., 2012), therefore, they seem to enjoy healthier social relationships.

The results of reliability analysis indicated that the Persian scale has an acceptable level of reliability, though fit indices were sub-optimal (although higher than the original ones). The results of inter-correlation analysis showed a significant positive correlation between the two subscales of the LMS.

## Conclusion

Based on the findings of the present study, it can be concluded that the Persian version of the LMS, with its two subscales and 10 items, enjoys good psychometric properties in the context of Iran. It is a reliable and valid tool that can be utilized for both social and clinical uses. It is also a short, easily-administered questionnaire that can be employed for research purposes. The existence of the Persian version of the LMS will help researchers conduct different correlational and experimental studies based on the socio-cognitive approach of mindfulness in the context of Persian speakers, given that the LMS is the only English-language instrument that is based on a sociocognitive view.

## Ethics statement

In order to collect the data, a permission letter was received from the municipality. To conduct the study passive consent was considered. Whoever who was not willing to participate was free to reject the researchers' request.

## Author contributions

The contribution of FM was in data collection, data analysis and writing the paper. The contribution of FP was in data analysis and writing the paper. The contribution of HK was in writing the paper.

## Funding

This research did not receive any specific grant from funding agencies in the public, commercial, or not-for-profit sectors.

### Conflict of interest statement

The reviewers AL and MK declared a shared affiliation, though no other collaboration, with the author FP to the handling Editor, who ensured that the process nevertheless met the standards of a fair and objective review. The authors declare that the research was conducted in the absence of any commercial or financial relationships that could be construed as a potential conflict of interest.
